# OX40–OX40L Axis in Cutaneous T-Cell Lymphomas: Pathogenic, Prognostic, and Potential Therapeutic Perspectives

**DOI:** 10.3390/biom15050715

**Published:** 2025-05-13

**Authors:** Alba Guglielmo, Alessandro Borghi, Corrado Zengarini, Bianca Maria Piraccini, Monica Corazza, Alessandro Pileri

**Affiliations:** 1 Section of Dermatology, Department of Medical Sciences, University of Ferrara, 44121 Ferrara, Italy; alessandro.borghi@unife.it (A.B.); monica.corazza@unife.it (M.C.); 2 Dermatology Unit, IRCCS Azienda Ospedaliero-Universitaria di Bologna, 40138 Bologna, Italy; corrado.zengarini2@unibo.it (C.Z.); biancamaria.piraccini@unibo.it (B.M.P.); alessandro.pileri2@unibo.it (A.P.)

**Keywords:** mycosis fungoides, Sézary syndrome, OX40–OX40L

## Abstract

Mycosis fungoides (MF) and Sézary syndrome (SS) are the most prevalent forms of cutaneous T-cell lymphoma (CTCL) and are characterized by the proliferation of CD4^+^ T-helper cells. The pathogenesis of CTCLs involves a critical interaction between neoplastic cells and the tumor microenvironment. This interaction is driven not only by cytokines but also by surface proteins that mediate cell–cell contact. One such protein, OX40 (also known as CD134), is a member of the TNF receptor superfamily and serves as an induced costimulatory molecule that facilitates the interaction between T-cells and antigen-presenting cells. In this narrative review, we explore the literature surrounding the OX40–OX40L interaction in CTCLs, highlighting its pathogenic and prognostic significance. Additionally, we compare the expression and function of OX40–OX40L in chronic inflammatory skin diseases, such as atopic dermatitis and psoriasis, with their role in CTCLs. Finally, we provide an overview of the current state of therapeutic research, discussing the potential of targeting the OX40–OX40L axis in CTCL treatment.

## 1. Introduction

Mycosis fungoides (MF) and Sézary syndrome (SS) are two types of cutaneous T-cell lymphoma (CTCL) characterized by pathological CD4^+^ T-cells [1]. MF is typically characterized by an indolent, slow-progressing course in its early stages, which can eventually evolve into a more aggressive and advanced form as the disease progresses. The clinical outcome of MF strongly correlates with the histological progression of the disease: in the early stages, skin lesions are marked by the presence of scattered neoplastic T-cells within a predominantly inflammatory background. As the disease advances, the skin lesions become more pronounced, and tumor lesions are largely composed of large blast cells that infiltrate the subcutaneous tissue, reflecting a shift towards a more aggressive disease. SS, a more advanced form of cutaneous T-cell lymphoma, is historically defined by the triad of erythroderma, generalized lymphadenopathy, and the presence of neoplastic T-cells known as Sézary cells in the skin, peripheral blood, and lymph nodes. The histopathological features of SS skin lesions are histologically indistinguishable from those seen in the patch and plaque stages of MF [2,3,4]. The biological mechanisms underlying this transition are not yet fully understood. Recently, various research groups have explored different aspects of the disease, including genomic abnormalities and changes in the tumor microenvironment [2,5,6]. In fact, the progression of the disease is driven by the interaction between neoplastic cells and the cells of the inflammatory “milieu” [7]. This interaction occurs both through the release of cytokines that can act at a distance from the producing cell, and through surface proteins that mediate cell-to-cell interactions, including those involved in antigen presentation and costimulation. The aim of this narrative review is to explore the mechanisms of cell-to-cell interaction in MF/SS, with a particular focus on the role of the OX40–OX40L axis. Furthermore, it is proposed to review the current understanding of the OX40–OX40L axis from a comparative perspective, focusing on CTCL and benign inflammatory dermatoses, particularly atopic dermatitis and psoriasis. Finally, some hypotheses regarding the potential use in CTCL of anti-OX40 targeted therapies will be presented.

## 2. Interactions Between CD4^+^ T-Cells and Antigen-Presenting Cells in Cutaneous T-Cell Lymphomas and Physiological Conditions: Similarities and Differences

CTCLs encompass a wide and heterogeneous group of clinicopathological entities, with each individual lymphoma being relatively rare.

Among these, MF and SS account for 70% of CTCL cases and appeared to be less frequent in North America (65%) compared to Europe (73%). The proportion of MF/SS among CTCL cases ranged from 40% in South Korea and Canada to 92% in Singapore [8,9]. In Italy, the exact epidemiology of CTCLs remains unknown; however, a national retrospective and prospective study is currently underway to gather data on their incidence and prevalence. The malignant cells in both MF and SS are CD4^+^ T lymphocytes, but they differ significantly between the two conditions. In SS, the malignant T-cells belong to the central memory T-cell subset and can circulate between the skin, lymph nodes, and blood. In contrast, the malignant T-cells in MF are non-recirculating, skin-resident effector memory T-cell [10]. This distinction is crucial and has enabled us to definitively differentiate MF, particularly its erythrodermic variant or advanced MF with nodal and blood involvement, from SS.

In physiological conditions, effector T-cells are those that actively engage in eliminating the infection or pathogen. Central memory T-cells, on the other hand, are a “reserve” form that ensures a faster and more effective response if the same antigen is encountered again in the future. CD4^+^ helper T-cells are restricted to MHC class II molecules and are activated only by antigen-presenting cells (APCs), and among them, Langerhans cells (LCs) and dendritic cells (DCs) play a pivotal role in cutaneous immunity. The primary function of helper T-cells is to secrete cytokines in response to antigenic stimulation, acting as “helpers” in both adaptive and innate immune responses. This contrasts with cytotoxic CD8^+^ T-cells, which directly kill target cells [11]. Antigens represent the first step in initiating the immune response and also play a crucial role in regulating specificity. In the T lymphocyte activation pathway, the term “antigen” specifically refers to protein-derived molecules that are processed and presented on MHC molecules. For the induction of antigen-specific T-cell-mediated immune responses, including anti-tumor immunity, antigen presentation by DCs is essential. Immature DCs capture antigens at peripheral sites, then undergo maturation and migrate to lymphoid organs, where they activate both helper and cytotoxic T-cells. LCs represent a subset of immature DCs located in the epidermis and are most notably characterized by Birbeck granules and can be identified through the expression of the langerin and LAG antigens. Upon maturation, DCs begin to express DC-LAMP and CD83, while displaying a reduced or absent expression of CD1a and CD1c, markers that are characteristic of their immature state.

Costimulation, or the second signal, which follows the first signal provided by the antigen, is a mechanism that enhances the T lymphocyte response after activation. The main costimulatory molecules in humans are the proteins B7-1 and B7-2. Both are membrane glycoproteins with an Ig domain in the extracellular portion and are expressed in APCs. B7-2 is expressed constitutively at low levels and is upregulated after activation, whereas B7-1 is not expressed initially but is induced hours or days after activation. Both B7-1 and B7-2 bind to the CD28 receptor on T lymphocytes [12]. Immature DCs, which have not yet encountered an antigen, express low levels of costimulatory molecules. Upon antigen encounter, these cells upregulate B7 expression, which is further sustained by the binding of CD40–CD40L with T lymphocytes [13]. This interaction plays a “licensing” role, stimulating APCs to express more costimulatory molecules and enhances their ability to activate other lymphocytes. APCs also maintain low levels of costimulatory molecules to regulate T lymphocytes and select autoreactive lymphocytes. When these lymphocytes strongly bind to the antigen but receive low levels of costimulation, they become anergic [14]. Additionally, APCs promote tolerance by generating T regulatory cells (T regs). T reg lymphocytes regulate immune responses by suppressing the activation and proliferation of various immune cells, including effector T-cells, B-cells, and DCs. T regs play a critical role in preventing excessive immune reactions and autoimmune diseases. Alongside these costimulatory molecules, we also recognize OX40–OX40L signaling.

## 3. A Focus on Antigen-Presenting Cells in Cutaneous T-Cell Lymphomas

To describe the role of APCs in CTCLs, the best starting point is likely the evaluation of the architecture of Darier’s nest (more commonly, though inaccurately referred to as Pautrier’s micro-abscess), which is the histopathological hallmark of MF. It is worth noting that the first to describe this histological hallmark was not Pautrier—who himself questioned the attribution of the eponym—but Darier [15]. Moreover, from a histopathological perspective, these epidermal collections of lymphocytes should not be referred to as “micro-abscesses”; the term “nests” is more accurate and appropriate [16].

Darier’s nest is characterized by the presence of multiple (at least four) atypical lymphocytes within a single epidermal vacuole [17], often accompanied by DCs [17,18,19]. These morphological and architectural features are consistent with the functional findings, particularly regarding the close interaction between lymphoma cells and APCs.

Numerous studies have characterized the expression levels of different APC subpopulations in CTCLs based on the lymphoma stage, and compared them to those in benign inflammatory diseases [20,21,22,23,24,25,26]. Langerin and DC-LAMP were used as markers for immature and mature DCs, respectively. Both markers showed a marked increase in the dermis of patients with CTCL compared to healthy skin [24]. In Lüftl et al.’s study, the mean number of langerin-positive cells per high-power field (40×) in the upper dermis was 1 in healthy controls, compared to 8 in patch lesions and 14 in tumor lesions. Similarly, the mean number of DC-LAMP-positive cells per high-power field was 1 in healthy controls, versus 10 in patch lesions and 15 in tumor lesions. All observed increases were statistically significant [22]. Moreover, in early-stage patch and plaque lesions, the epidermis also contains a high number of immature DCs that express langerin antigens [20]. In this area, langerin-positive cells were mainly located within the infiltrate of T lymphoma cells and tended to form clusters [24]. In tumor lesions, the number of DCs in the dermis is even higher than in early MF lesions. Numerous DCs, both immature and mature, are interspersed within the dermal infiltrate. The immature DCs found in the lower dermis of tumor lesions do not co-express langerin or LAG, suggesting that they may not be derived from LCs [22]. The authors suggest that in early MF, immature DCs from the epidermis mature and migrate to the papillary dermis, where they function as highly efficient APCs in areas of epidermotropic tumor T-cell invasion [19,22]. This may explain why an increased number of LCs can be observed both in the dermal component and within the epidermis [19]. Schwingshackl et al. found that BDCA-2-positive plasmacytoid DCs were increased in both MF and SS compared to the skin of healthy donors. These cells were irregularly distributed throughout the dermal infiltrate, with a few positively stained cells also present in the lower part of the epidermis [24]. Pileri et al. observed that plasmacytoid DCs were more abundant in tumor lesions of MF compared to patch and plaque lesions [20]. Schlapbach et al. analyzed the expression and tissue distribution of various DC subset markers in lesional and non-lesional skin of patients with CTCL. They found significant infiltration of CTCL lesions by immature CD209/DC-SIGN^+^ DCs, which were in close contact with tumor cells. Similar results were observed for CD303/BDCA-2, a marker of plasmacytoid DCs, which was upregulated, though overall expression was infrequent. In contrast, mature and activated DCs were rarely detected in CTCL lesions. The authors also analyzed different subsets of T-cells in lesional skin, focusing on T reg lymphocytes by evaluating the immunostaining of FoxP3 and CD25 markers. A significant increase in T regs was observed in the dermis, and the expression pattern of T reg markers correlated with the expression pattern of immature interstitial DC markers. Moreover, T reg cells were found in close contact with immature DCs [26]. In accordance with a previous study by Berger, CTCL cells can adopt a T reg phenotype and function after stimulation by immature DCs. These T regs were shown to secrete IL-10, maintaining DC immaturity and consequently promoting the induction of further T regs [27]. The resulting accumulation of immature DCs and malignant T regs would create an immunosuppressive environment, thereby facilitating tumor growth and immune escape. Cioplea et al. investigated the differences DC expression between cutaneous lymphomas (predominantly MF) and various types of cutaneous inflammatory diseases. Immunohistochemical analysis was conducted using the markers CD1a, CD11c, and langerin. The first significant finding was of a “quantitative” nature, as DCs were present in greater numbers within the neoplastic infiltrate compared to benign inflammatory dermatoses. Additionally, the distribution pattern of DCs varied. In benign inflammatory dermatoses, DCs predominantly exhibited a “nodular” distribution, while in lymphoma cases, the most common patterns were “arachnoid” distribution and interspersed with tumor cells. The authors proposed that the differences in morphological distribution could be correlated with the distinct functional roles of DCs in inflammatory versus neoplastic conditions. Specifically, in benign inflammatory dermatoses, DCs play a role in activating and modulating the inflammatory response; whereas in CTCL, they not only contribute to disease initiation but may also be involved in its progression or eventual regression [25].

APCs in CTCL perform a dual role: they induce and maintain antitumor immunity, or, under less favorable conditions such as IL-10 production, they downregulate antitumor immunity. All authors agree that the accumulation of immature DCs in MF samples is consistent with a shift from a Th-1 to a Th-2 phenotype, and that the abundance of immunosuppressive cytokines released by neoplastic and “milieu” cells ultimately creates a tolerogenic microenvironment that promotes tumor progression [5,20,28]. CTCL progression is marked by a shift in cytokine profiles from a Th1 to a Th2 dominance [29]. Several studies have shown that Th1 cytokines, including IFN-γ, IL-2, and IL-12, are predominantly expressed in early-stage MF. In contrast, tumor cells in advanced-stage MF and SS lesions exhibit a Th2 phenotype, with elevated expression of Th2-associated cytokines, such as IL-4, IL-5, IL-10, and IL-13 [30]. At the gene expression level, advanced stages are associated with increased expression of GATA3 [31]. Using T-bet as a specific marker of Th1 cells and GATA3 as a marker of Th2 cells, it has been observed that T-bet expression is elevated and predominant in early MF [29]. However, as the disease progresses, GATA3 expression gradually increases and eventually becomes dominant [31].

Regarding CD4^+^ T-cell subsets, OX40–OX40L signals can enhance the Th1-mediated immune response and promote both the generation and maintenance of Th2 cells [32]. We can hypothesize that the OX40–OX40L axis may play a role in both the early stages of MF and in the advanced stages of the disease and SS.

## 4. Role of the OX40–OX40L Axis in Cutaneous T-CELL Lymphoma and Benign Inflammatory Dermatoses

OX40, also known as CD134, is a surface protein expressed on activated CD4^+^ T-cells, natural killer cells, and T reg cells. OX40 is a member of the TNF receptor (TNFR) superfamily and is a type I transmembrane protein [33,34]. Its ligand, OX40L, is expressed by APCs, including LCs, DCs, and activated B-cells. TNFR-related proteins are crucially involved in the regulation of the proliferation and survival of normal and malignant lymphohematopoietic cells. In particular, OX40–OX40L signaling activates several downstream signaling pathways, including the PI3-kinase/AKT pathway, the FAS pathway, NF-κB, and BCL-2, all of which promote T-cell proliferation and survival.

OX40 expression can be induced in various T-cell subsets, including CD4^+^ and CD8^+^ T lymphocytes, natural killer (NK) cells, and T reg cells. Interestingly, OX40–OX40L interaction can induce or modulate a range of immune responses depending on the cytokine “milieu” to which T lymphocytes and APCs are exposed. In this regard, an overview of OX40–OX40L activity in physiological conditions or inflammatory disease is presented below.

OX40–OX40L interactions play a key role in enhancing ongoing immune responses, contributing to both Th1- and Th2-type polarization [35,36]. This is supported by studies in animal models, where mice deficient in either OX40 or OX40L exhibit reduced Th1 and Th2 cytokine responses in vivo. OX40–OX40L signaling, in the presence of cytokines such as IL-4 and IFN-γ, promotes the preferential expansion of effector CD4^+^ T-cell populations. Conversely, in the absence of IL-4 and IFN-γ, the same signaling pathway favors the expansion of T regs [37]. These findings suggest that in a proinflammatory microenvironment, OX40 signaling amplifies immune responses via the action of inflammatory cytokines, whereas in the absence of such signals, it fosters a more immunoregulatory phenotype [37]. Moreover, when naive T lymphocytes are exposed to IL-4, they differentiate into Th2 cells; in this case, OX40 interaction promotes differentiation towards Th2 and the generation of memory T-cells [36].

**Figure 1:**
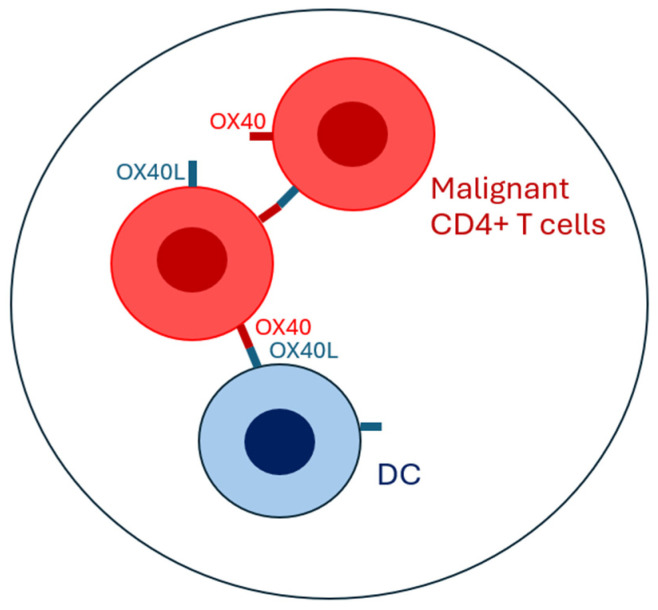
Diagrammatic representation of the cellular crosstalk between dendritic cells (DCs) and malignant CD4^+^ T lymphocytes in mycosis fungoides, with a focus on OX40–OX40L-dependent signaling pathways.

OX40 also modulates the function and survival of Th17 lymphocytes in both activating and inhibitory manners [38,39]. Finally, OX40 acts as a promoter of inflammatory and immune effector responses by stimulating CD8^+^ T lymphocytes, antagonizing the production and suppressive activity of T regs, and stimulating other costimulatory pathways, including ICOS, which enhance the generation of T follicular helper lymphocytes [40,41,42]. In MF/SS, lymphoma CD4^+^ T-cells express not only OX40 but also OX40L. Kawana et al. compared the expression levels of OX40 and OX40L mRNAs between healthy and lesional skin, revealing significantly higher expression in lesional skin, particularly in patients with advanced disease. Moreover, the expression levels of OX40 mRNA correlate with those of OX40L mRNA. These findings are further supported by immunohistochemical staining results [43]. Additionally, elevated levels of the OX40 and OX40L are associated with a worse prognosis and increased disease-specific mortality. Although OX40L expression is predominantly observed in APCs, the authors noted that in circulating CD4^+^/CD7^−^ Sézary T-cells, there was an increased expression of both OX40 and OX40L. This contrasts with what is observed in healthy individuals, where circulating CD4^+^ T-cells express OX40 and only weakly express OX40L. This observation suggests that the co-expression of OX40 and OX40L on lymphoma T-cells, and the subsequent activation of the OX40–OX40L axis, may contribute to the onset and progression of MF and SS [43] (Figure 1). These observations are only partially supported by data published in a previous study by Jones et al. The authors found that in the skin of five patients with MF, OX40 expression was rarely observed, with reactivity to staining seen in fewer than 5% of tumor cells. However, when lymph node involvement was observed in cases of MF with large cell transformation, OX40 staining was expressed in three out of four cases [44]. One of the limitations of the observations made by Jones et al. is the small number of MF cases. However, their study already highlighted an increase in OX40 expression, which was correlated with more advanced stages and a worsening prognosis.

It is noteworthy that the expression patterns of the TNFR superfamily play a critical role in the immunophenotypic subclassification of lymphomas, including primary cutaneous lymphomas. A prime example is CD30, a 120 kDa type I membrane protein, which has been extensively studied and has become integral to the nomenclature of primary cutaneous CD30-positive lymphoproliferative disorders, such as lymphomatoid papulosis (LyP), anaplastic large cell lymphoma (ALCL), and transformed MF [45].

Gniadecki and Rossen conducted an immunohistochemical study to assess OX40 expression levels in CTCL and benign inflammatory dermatoses, including cases of LyP, MF, Jessner’s infiltrate, and non-specific dermatitis. The findings from this study contrast with those of previously described studies. Specifically, a strong OX40 expression was observed in a subset of patients with LyP (38% of cases), whereas OX40 expression in MF and benign inflammatory dermatoses biopsies was low, involving only single scattered cells [46]. In the same study, it was observed for the first time that OX40 was co-expressed with CD30 on atypical cells. Finally, in the only patient who showed progression of MF, with large cell transformation and lymph node involvement, no change in the pattern of OX40 expression was observed, raising questions about the prognostic significance of OX40 [46]. The most recent study by Kawana et al. [43], based on a larger case series, appears to confirm the correlation between increased OX40 expression and progression to more advanced stages of lymphoma, as previously hypothesized by Jones et al. [44].

The OX40–OX40L axis is recognized to play a pivotal role in benign inflammatory diseases, such as AD and psoriasis. Activation of the OX40–OX40L axis is just one of many factors involved in the pathogenesis of AD. In fact, skin barrier dysfunction, skin microbiome dysbiosis, and dysregulation of the immune response towards Th2 polarization during the acute phases are the main mechanisms underlying AD. OX40–OX40L signaling facilitates the differentiation of Th2 cells [47,48]. Cytokines such as IL-25 and IL-33, which drive type 2 inflammation, further enhance OX40L expression. The engagement of OX40 on activated CD4^+^ T-cells by OX40L leads to the expansion of effector T helper cells, primarily Th2 cells, that produce early pathogenic cytokines such as IL-4 and IL-13 [48]. While, in chronic phases of AD, sustained OX40–OX40L signaling supports the proliferation of additional effector T-cell subsets, including Th1, Th17, and Th22 cells. These cells upregulate the production of proinflammatory cytokines such as IFN-γ, IL-17, and IL-22 [48]. In CTCL, a similar but inverse mechanism is observed. During the early stages, OX40–OX40L signaling likely promotes a Th1-skewed response, while in advanced disease, the pathway shifts to favor a Th2-dominant immune profile [2,7].

OX40 modulates the survival and function of Th17 cells, exerting opposing effects depending on the context and disease. For example, in an experimental model of arthritis [38], the production of IL-17 from activated T-cells is required for the spontaneous development of destructive arthritis in mice deficient in the IL-1 receptor antagonist. In uveitis [39], the activation of OX40 enhances Th17 cytokine expression and promotes uveitis development. Conversely, other studies have shown that the binding of OX40 with its ligand leads to the upregulation of IFN-gamma and IL-4, both of which inhibit IL-17 production, even in the presence of IL-23 [49]. Additionally, this interaction induces the accumulation of T regs, which exert a suppressive effect on the immune response [50].

Guo et al. conducted a study on circulating T lymphocytes in psoriasis patients, identifying a CD3–CD4^+^ subset with high expression of OX40, while OX40L was rarely expressed in these cells. Regarding the skin expression of OX40, it was found to be higher in lesional than in non-lesional skin [51]. Finally, Ilves and Harvima observed the expression of the OX40 and OX40L in the lesional skin of AD patients, with higher levels compared to non-lesional skin and psoriasis. However, no correlation was found between OX40 and OX40L expression levels and AD severity [52] (Table 1).

## 5. Future Perspectives and Therapeutic Opportunities

The treatment approach for CTCL is primarily guided by the stage of the disease, which remains the most significant prognostic factor. Treatment plans are designed based on the compartments affected by the disease, particularly whether the disease is restricted to the skin or has spread to other areas, such as the lymph nodes, internal organs, or bloodstream. In early-stage CTCL (stages IA–IIA), where the disease is confined to the skin, treatment generally focuses on therapies directed at the skin. These include topical medications like corticosteroids, other topical chemotherapies, phototherapy, or localized radiation. These methods are effective in managing localized disease and have minimal cumulative toxicity, making them the preferred treatment for patients at this stage. The goal in early-stage CTCL is to alleviate symptoms, reduce overall disease burden, and improve the patient’s quality of life with as few side effects as possible. As the disease progresses to later stages (stages IIB-IVB), where the disease either becomes resistant to initial treatments or starts to affect other parts of the body, including the lymph nodes, internal organs, or blood, systemic therapies are introduced. These systemic treatments include drugs such as bexarotene, interferon-alpha, oral methotrexate, extracorporeal photopheresis, pralatrexate, and histone deacetylase inhibitors like romidepsin and vorinostat. These therapies have primarily been evaluated in phase II clinical trials and observational studies, showing effectiveness in managing the disease, although they do not offer a cure [1,53,54].

Recently, more effective therapies have been developed, particularly brentuximab vedotin (BV) and mogamulizumab, both of which have been approved based on results from phase III clinical trials. These newer therapies have demonstrated higher response rates and more durable outcomes compared to traditional treatments. Despite this progress, however, no treatment provides a definitive cure, and many patients with advanced or refractory disease will require multiple rounds of systemic treatment to manage the disease [1,55].

In addition to the standard therapies, several new agents are currently being tested in clinical trials. These include dimethyl fumarate (DMF), lacutamab, PD-1 inhibitors (such as pembrolizumab and tislelizumab), and ruxolitinib [54]. Early results have shown these treatments to be promising, with encouraging clinical responses. These newer therapies often target different immune pathways or tumor microenvironments, offering hope for more effective treatments and potentially better outcomes, especially in patients with advanced or resistant diseases. At the same time, cancer immunotherapy has significantly advanced the treatment of various cancers by leveraging the immune system to specifically target and destroy tumor cells. While checkpoint inhibitors have been successful in many types of cancer, agonist antibodies targeting costimulatory receptors, such as ICOS, GITR, OX40, CD27, and 4-1BB, are currently under investigation [53].

This suggests that despite the potential of agonist antibodies to stimulate the immune response, their efficacy has been limited, necessitating new strategies and combinations to overcome their shortcomings and improve clinical outcomes for patients with CTCL.

This review aims to explore the current understanding of the OX40–OX40L axis in CTCL and compare it with benign inflammatory dermatoses. Although studies are limited, the available evidence suggests that and its ligand may have prognostic significance in patients with MF and SS. Additionally, the expression of OX40 and its ligand appears to differ between CTCL and benign inflammatory dermatoses, particularly in terms of expression levels or patterns. OX40L is typically expressed on APCs under physiological conditions and in benign inflammatory dermatoses. However, in CTCL, neoplastic T-cells express both OX40L and OX40. Further immunohistochemical studies are needed to confirm this association on a larger scale. If validated, the evaluation of OX40L expression could serve as an immunohistochemical marker to assist in the histological diagnosis of early MF or SS and help differentiate them from benign inflammatory dermatoses in ambiguous cases.

Several emerging therapies targeting and inhibiting the OX40–OX40L pathway are currently in development. In AD, the monoclonal antibodies anti-OX40 rocatinlimab (NCT03703102) and telazorlimab (NCT03568162), as well as the anti-OX40L monoclonal antibody amlitelimab (NCT05131477) are currently under investigation. Additionally, rocatinlimab is currently being developed for other indications, as demonstrated in patients with psoriasis [51]. Currently, there are no clinical studies investigating the use of OX40–OX40L inhibitors in cancer, although the role of OX40 has been demonstrated in the adhesion mechanisms of neoplastic cells to the endothelium [56] and in skin infiltration observed in adult T-cell leukemia [57].

The inhibition of OX40 and OX40L has been shown to suppress tumor cell proliferation, with the OX40-neutralizing antibody inducing apoptosis in CTCL cells. Kawana et al. evaluated the in vivo effects of anti-OX40 and anti-OX40L neutralizing antibodies in a tumor dissemination model using immunodeficient mice. The results indicated a significant suppression of tumor formation in the groups administered with anti-OX40 and anti-OX40L antibodies compared to the control group. These findings led the authors to suggest that anti-OX40 and anti-OX40L antibodies could represent promising therapeutic options for MF and SS. On the other hand, the OX40 signaling pathway enhances anti-tumor immune responses in various malignancies by stimulating immune cells [43]. Consequently, OX40–OX40L agonists are currently being investigated in clinical trials for several cancers, including non-small cell lung cancer, squamous cell carcinoma of the head and neck, malignant melanoma, triple-negative breast cancer, and colorectal cancer [58,59,60,61,62]. However, the effects of such immunotherapies have shown considerable variability across patients, with therapeutic responses observed only in a subset of individuals. It is important to note that CTCLs differ significantly from other malignancies. In CTCL, CD4^+^ T-cells represent not only the neoplastic population but also part of the tumor-infiltrating lymphocytes (TILs) that contribute to the inflammatory environment. Therefore, while inhibiting the OX40–OX40L axis may hinder the growth of neoplastic cells, such an approach could inadvertently promote tumor progression if it affects TILs. Furthermore, while OX40 agonists could potentially enhance anti-tumor responses, there are risks associated with stimulating tumor cell growth, particularly in the context of MF/SS. As such, treatment strategies targeting the OX40–OX40L pathway in CTCL need to carefully balance the enhancement of immune responses with the potential for adverse effects on tumor progression. The treatment of advanced CTCL remains challenging despite the development of new therapies. Currently, no clinical trials are evaluating the use of anti-OX40 or anti-OX40L therapies in MF or SS, nor have any case reports or small patient cohorts been documented in the literature. Available data are limited to preclinical studies conducted in murine models [43]. As observed in other tumor types, OX40–OX40L-targeting drugs may potentially be used as monotherapy or in combination with immunotherapy, although the agents studied so far have primarily acted as agonists of the OX40–OX40L axis [63]. Inhibitors of OX40–OX40L may also hold therapeutic promise in CTCL; however, their application will likely be limited to selected patients exhibiting high OX40–OX40L expression on tumor T-cells. At the same time, appropriate assessments should also be conducted on TILs and APCs within the tumor microenvironment in CTCL. OX40 and OX40L expression can be evaluated using various methods, including immunohistochemistry and mRNA expression analysis. In this context, OX40–OX40L agonists that stimulate the anti-tumor immune response may represent a promising therapeutic strategy; however, in the future, it will be essential to balance this effect against the potential risk of disease progression in cutaneous lymphomas.

## 6. Conclusions

MF/SS are T-cell lymphomas in which the tumor microenvironment plays a critical role in disease progression. While cytokine expression has been extensively studied, less is known about direct cell–cell interactions. The OX40–OX40L axis is a key costimulatory pathway that mediates the interaction between malignant T-cells and dendritic cells. In this review, we summarize the current knowledge regarding OX40 and its ligand in MF/SS, and compare the underlying mechanisms with those described in chronic inflammatory skin disorders. A deeper understanding of these interactions, along with future clinical trials, may pave the way for novel therapeutic strategies for patients with CTCL.

## Figures and Tables

**Table 1:** The role of the OX40–OX40L axis in cutaneous T-cell lymphoma and benign inflammatory dermatoses.

	OX40 Expression Compared to Healthy Skin	OX40L Expression Compared to Healthy Skin	Pathogenetic Mechanism	Prognostic Significance of OX40–OX40L Axis
**Cutaneous T-Cell Lymphoma**	Increased	Increased	In early MF: increased Th1, cytokine expression.In advanced MF and SS: increased Th2 and Treg cytokine expression	Increased disease-specific mortality (Kawana et al. [43])
**Atopic dermatitis**	Increased	Increased	In acute phase: increased Th2 cytokine expression.In chronic phase: increased Th1, Th17, Th22 cytokine expression	No correlation with AD severity (Ilves and Harvima [52])
**Psoriasis**	Increased	Low	Increased Th17 cytokine expression	Not known (Guo et al. [51])

## Data Availability

No new data were created or analyzed in this study.
